# Experimental and Modeling Assessment of Polyphenol Solubility in Alcohol + Ethyl Acetate Mixtures for Extraction Applications

**DOI:** 10.1021/acsomega.5c10953

**Published:** 2026-02-26

**Authors:** Iván Montenegro, Begoña González, Ángeles Domínguez, Elena Gómez

**Affiliations:** FEQx Lab, Department of Chemical Engineering, 16784 University of Vigo, Vigo 36310, Spain

## Abstract

Polyphenols are valuable bioactive compounds widely used in the pharmaceutical and cosmetic industries, yet their extraction is often limited by low solubility in conventional solvents. This work extends the solubility database of four polyphenols (*trans*-polydatin, *p*-coumaric acid, quercetin, and *trans*-resveratrol) by determining and modeling their equilibrium solubility in methanol, ethanol, and ethyl acetate at 298.2 K and 0.1 MPa. Additionally, solubility in two binary mixtures (methanol + ethyl acetate and ethanol + ethyl acetate) was measured to assess the ester cosolvent effect. Excess solubility was calculated to evaluate nonideality, and experimental data were correlated using the Abraham solvation model, the CNIBS/R–K equation, and solvatochromic (KAT) parameters. Higher solubility was observed in ethanol than methanol for all compounds except for *trans*-polydatin, while ethyl acetate showed the lowest values. All ternary systems exhibited solubility maxima due to synergistic effect of ethyl acetate, being the greatest solubility enhancement observed for *trans*-resveratrol, with a 129% increase at 0.4 mole fraction of the ester solvent in the methanol + ethyl acetate binary solvent mixture. Eventually, all systems were accurately described by the Abraham model, CNIBS/R-K equation, and KAT parameters.

## Graphical abstract

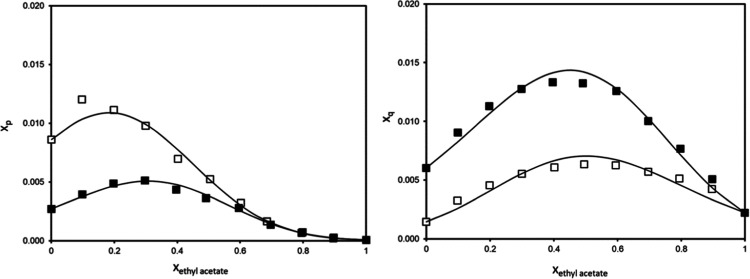

## 1 Introduction

Among the wide variety of phenolic compounds, polyphenols from natural sources stand out due to their antioxidant activity and associated biological benefits. Consequently, they have been attracting considerable interest in recent years for their potential application in medicine and pharmaceutics, including disease treatment, antiaging strategies, and anticancer therapies.^1–8^

The sustainable extraction of these compounds is therefore paramount in several industrial sectors, thus requiring careful solvent selection. This may constitute a challenging task, due to the wide variety of polyphenols present in natural sources and the large number of solvents available for extraction processes.^9–14^ Methanol, ethanol, and their aqueous mixtures are the most commonly used, leading to good results regarding the total phenolic content (TPC) of the extract;^15–25^ in addition, they are easy to recover through conventional vacuum distillation, whereas other approaches such as the use deep eutectic solvents (DES) highly hinders the eventual purification of polyphenols from the extraction medium. However, a sequential extraction must be performed if selectivity is pursued, employing different solvent combinations. For this reason, carrying out preliminary solubility studies of polyphenols is highly advisable.

Solubility analysis of pharmaceutical compounds is crucial for the development of crystallization, purification, and extraction processes.^26^ As a result, determining the solubility of a specific polyphenol in a wide range of solvents will help to establish an effective strategy for the efficient recovery of the target compound. Although the use of pure solvents yields good results for extraction, binary mixtures of organic solvents are being increasingly studied for providing better solubilities. Many investigations have experimentally demonstrated that the solubility of certain compounds in binary solvent mixtures increases relative to that in the corresponding pure solvents, achieving a maximum value at a specific solvent composition.^27–37^ This phenomenon, commonly referred to as the “maximum solubility effect”, is due to the synergistic behavior of the solvents in favor of solute solvation, which can be strategically used to develop selective separation sequences in the industrial sector.

In this regard, predictive models have become key tools to develop efficient extraction systems.^38–41^ These models, whether based on theoretical principles or data-driven methods, enable the estimation of solubility under multiple conditions, promoting solvent screening and separation process optimization while reducing experimental workload; in addition, robust models can contribute to a deeper comprehension of molecular interactions. Likewise, experimental determination of solubility data in wide ranges of pure solvents and multicomponent solvent mixtures remains essential for corroborating the results yielded by predictive models and thus thoroughly understanding solute–solvent interactions.

In one of our previous articles, we determined the solubility of five polyphenols in four different organic monosolvents, as well as that of two polyphenols in three binary solvent mixtures.^42^ The results revealed a solubility enhancement in both binary solvent mixtures for the two studied polyphenols. Based on that work, and with the aim of further extending that research by covering more polyphenols and solvents, in this study we measured the equilibrium solubility of four compounds from different polyphenolic subclasses (*trans*-polydatin, *p*-coumaric acid, quercetin, and *trans*-resveratrol) in pure ethanol, and *p*-coumaric acid also in methanol and ethyl acetate at 298.2 K and 0.1 MPa. Additionally, the solubility of those polyphenols in ethanol + ethyl acetate along with that of *p*-coumaric and *trans*-resveratrol in methanol + ethyl acetate was determined under the same conditions to evaluate the synergistic effect of the ester solvent. Solubility excesses were calculated, experimental solubility in pure solvents was correlated using the Abraham solvation model, and that in binary mixtures was regressed with the combined nearly ideal binary solvent/Redlich–Kister (CNIBS/R–K) equation. Eventually, the solvent effect was analyzed using the solvatochromic parameters α, β, and π* and the Hildebrand solubility parameter δ_H_^2^.

## 2 Methodology

### 2.1 Chemicals

Mass purity, CAS number, molecular weight, and suppliers of the reactants used in this study are reported in Table 1. The selection of all polyphenols (see the structure in Figure S1) was based on their application and abundance in natural sources, whereas the solvents were chosen according to their green character and chemical affinity with the polyphenols. More specific information regarding the selection criteria of the compounds can be found in our aforementioned article.^42^

**Table 1:** Information of materials used for experimentation

compound	CAS number	source	molecular weight/g·mol^–1^	mass purity^a^/%	purification method
*trans*-polydatin	65914–17–2	sigma-aldrich	390.38	≥95	drying^b^
*p*-coumaric acid	501–98–4	sigma-aldrich	164.16	≥98.0	drying^b^
quercetin	117–39–5	sigma-aldrich	302.24	≥95	drying^b^
*trans*-resveratrol	501–36–0	TCI	228.24	>99.0	drying^b^
methanol	67–56–1	sigma-aldrich	32.04	≥99.9	N.A^c^
ethanol	64–17–5	sigma-aldrich	46.07	≥99.5	N.A^c^
ethyl acetate	141–78–6	sigma-aldrich	88.11	≥99.5	N.A^c^

a Provided by the supplier, with water content <0.2%.

b At 323.25 K for 3 h.

c Not applicable.

All solvents were used without extra purification and polyphenols were dried at 323.15 K for 3 h before their use, ensuring that no thermal degradation of the compounds occurred.^8^

### 2.2 Experimental Procedure

#### 2.2.1 Stability Study by UV–Vis Spectroscopy

Polyphenols can exist as different charged species due to their high reactivity. In order to perform an accurate quantification of the solutes, their chemical stability must be guaranteed in all solvents. To ensure it, stability of *trans*-polydatin, *p*-coumaric acid, quercetin, and *trans*-resveratrol in methanol, ethanol, and ethyl acetate with time was evaluated via Ultraviolet–Visible (UV–vis) spectrophotometry with a JASCO V-750 spectrophotometer (STA 449F3, Netzsch, Germany).

#### 2.2.2 Solubility Determination in Pure and Binary Solvent Mixtures

The solubility of *trans*-polydatin, *p*-coumaric acid, quercetin, and *trans*-resveratrol in pure ethanol and the solubility of *p*-coumaric acid in pure methanol and ethyl acetate was determined by the equilibrium method at a constant temperature of 298.2 K and 0.1 MPa. This same procedure was applied to measure the solubility of those polyphenols in binary solvent mixtures containing ethanol + ethyl acetate and that of *trans*-resveratrol and *p*-coumaric acid in the methanol + ethyl acetate binary solvent mixture, covering the whole molar fraction range. The procedure can be summarized as follows: an excess of solute is added to 0.5 mL of pure solvent or binary mixture in 1.5 mL Eppendorf tubes, then samples are agitated with a magnetic stirrer in a water bath at 298.2 K for 2 h and centrifuged to separate both phases at 12,000 rpm for 20 min. A 0.1 mL aliquot of the supernatant is extracted, weighed, and left to dry until total solvent evaporation. Dry residues are finally redissolved and diluted in methanol, so that the measured absorbance values are within the calibration range.

Solubility was determined by UV–vis spectrophotometry, and then calibration curves of each polyphenol in methanol (see Figure S2 in Supporting Information) were performed at the maximum wavelength depending on the solute (at 305 nm for *trans*-polydatin and *trans*-resveratrol, at 311 nm for *p*-coumaric acid, and at 370 nm for quercetin).

A more detailed description of the experimental procedure is provided in a previous article,^42^ which also includes the verification of the applied method for solubility determination.

#### 2.2.3 Powder X-ray Diffraction

X-ray diffraction studies were conducted to characterize the crystalline structures of *trans*-polydatin, *p*-coumaric acid, quercetin, and *trans*-resveratrol after recrystallization in pure solvents. Samples were obtained after solvent evaporation from saturated solutions at room temperature, and PXRD analysis of raw polyphenols and recrystallized samples was performed using an X-ray diffractometer (X’PERT PRO, PANalytical, Netherlands), with a 40 kV voltage and 30 mA current. The scanning angle was set from 4.5° ≤ 2θ ≥ 60°, and the step size was 0.026°.

### 2.3 Data Treatment

#### 2.3.1 Abraham Solvation Model

The Abraham solvation model is a widely used method to effectively describe the solubility behavior of molecules in pure solvents, being formulated within the framework of linear free energy relationships (LFERs). The logarithm of the experimental solute partition (in molar concentration basis) in an organic solvent (*C*_s_) and in water (*C*_w_) is correlated with eq 1.^43^

1 $$\log\left(\frac{C_{s}}{C_{w}}\right)=c+eE+sS+aA+bB+vV$$

where the uppercase parameters (E, S, A, B, and V) represent the Abraham descriptors of the solute and the lowercase parameters (c, e, s, a, b, and v) are referenced to the organic solvent.

Abraham descriptors capture key physicochemical properties of both the solute and the solvent: E represents the excess molar refraction, in relation with the “π” electron and n-electron interactions associated with unsaturated and aromatic systems, whereas *S* accounts for dipolarity and polarizability effects. For its part, A and B quantify the ability of the compound to act as a hydrogen bond donor or acceptor, respectively, reflecting the acid or basic behavior of the molecule. Eventually, *V* corresponds to McGowan’s characteristic volume, a measure of molecular size and dispersion interactions of the solute, as well as the cavity formation tendency of the solvent.

All Abraham descriptors of solutes and solvents were taken from the UFZ-LSER database^44^—except those of *p*-coumaric acid^45^—and are presented in Tables S1 and S2 of the Supporting Information, with no extra calculations nor modifications from our side. Since descriptors of *trans*-polydatin have not been reported in the literature to date, an attempt to calculate them was made using a reduced set of LFERs (eq 1) to regress the experimental solubility data determined in this work; however, no convergence was achieved for any of the tested sets, and thus solubility of *trans*-polydatin could not be correlated with the Abraham solvation model.

Mole fraction solubility of *p*-coumaric acid, quercetin, and *trans*-resveratrol in water was experimentally determined at 298.2 K and 0.1 MPa following the procedure described in the previous section, yielding values of 2.376·10^–6^, 6.279·10^–5^, and 4.701·10^–6^, respectively. Experimental mole fraction solubility values of the three polyphenols in methanol, ethanol, ethyl acetate, and water were converted to the molar basis, the experimental partition was calculated, and Abraham descriptors were used to predict them using eq 1.

#### 2.3.2 Excess Solubility

Solubility excess can provide useful information about the synergistic or antagonistic mixing effects among solvents in a multicomponent mixture. To gain a deeper understanding of solubility behavior of polyphenols in binary solvent mixtures, experimental solubility data were further analyzed in terms of deviation from ideality using eq 2.

2 $$\ln x^{E}=\ln x-\left[x_{1}\ln\left(x_{1}^{0}\right)+x_{2}\ln\left(x_{2}^{0}\right)\right]$$

where *x* ^E^ is the solubility excess, *x* is the mole fraction solubility of a certain polyphenol in a binary solvent mixture with a *x*_1_ mole fraction of alcohol and *x*_2_ mole fraction of ethyl acetate, and *x*_1_^0^ and *x*_2_^0^ are the mole fraction solubilities of the compound in pure alcohol and ethyl acetate, respectively.

#### 2.3.3 CNIBS/R–K Solubility Model

The combined nearly ideal binary solvent/Redlich–Kister equation (CNIBS/R–K)^46^ is a common thermodynamic model for excess solubility data fitting concerning binary solvent mixtures under isothermal conditions, having proven to show high predictability in numerous experimental studies.^40–42^ It relies in the following expression

3 $$\ln x=x_{1}\ln\left(x_{1}^{0}\right)+x_{2}\ln\left(x_{2}^{0}\right)+x_{1}x_{2}\sum_{i=0}^{n}S_{i}{\left(x_{2}-x_{1}\right)}^{i}$$

where *x* is the mole fraction solubility of a given polyphenol in a binary mixture with a *x*_1_ mole fraction of alcohol and *x*_2_ mole fraction of ethyl acetate, *x*_1_^0^ and *x*_2_^0^ are the mole fraction solubilities of the compound in pure alcohol and ethyl acetate, respectively, and *S*_*i*_ stands for the model constants with *n* = {0, 1, 2, 3}.

This equation was applied to the six experimental solubility data sets obtained in this work, namely those concerning *trans*-polydatin, *p*-coumaric acid, quercetin, and *trans*-resveratrol in the binary solvent mixture containing ethanol + ethyl acetate, and those regarding *p*-coumaric acid and *trans*-resveratrol in methanol + ethyl acetate binary solvent mixture.

#### 2.3.4 Kamlet–Taft Solvatochromic Parameters

To evaluate the influence of intermolecular solvent–solvent and solute–solvent interactions on the solubility of the four polyphenolic compounds in the two binary mixtures studied (ethanol + ethyl acetate and methanol + ethyl acetate), the solvatochromic parameters α, β, and π* and the Hildebrand solubility parameter δ_H_^2^ were analyzed. The α parameter represents the acidity of the hydrogen bonds between the solute and the solvent, reflecting the solvent’s ability to donate a proton in the solute–solvent hydrogen bond. The β parameter denotes the basicity of the hydrogen bonds between the solute and the solvent, reflecting the solvent’s capacity to accept a proton. The π* parameter corresponds to the solvent polarizability index, which quantifies its ability to stabilize a charge or a dipole due to its dielectric effect. Finally, δ_H_^2^ describes the energy required to overcome the attractive intermolecular forces between solvent molecules in order to form a cavity of appropriate size to accommodate the solute.^49^

The linear solvation energy relationship (LSER) model is a widely applied approach for describing solvent effects in terms of intermolecular interactions. Based on this framework, Kamlet, Abboud, and Taft developed the KAT-LSER model,^50^ which correlates solubility with the solvatochromic parameters (α, β, and π*) and whose results can be adjusted using multiple linear regression analysis (MLRA). The classical expression of the KAT-LSER model is presented in eq 4.^50^

4 $$\ln\left(x\right)=C_{0}+C_{1}\alpha +C_{2}\beta +C_{3}\pi^{*}+C_{4}\left(\delta_{H}^{2}/1000\right)$$

where *x* indicates the molar fraction solubility, α, β, and π* are the KAT parameters; and the last term of the equation, δ_H_^2^, denotes the Hildebrand solubility parameter of the solvent. The coefficients (*C*_*i*_, where *i* = 0, 1, 2, 3, and 4) represent the relative contributions of each interaction to solubility.

Considering the representation of our solubility values as a function of composition for our systems, it was concluded that a simple linear correlation of ln (*x*) with the KAT parameters is not sufficient to adequately describe the experimental behavior. This limitation arises from the complexity of the solute–solvent interactions involved, which cannot be fully captured by a purely linear model. Therefore, in this work, we explored alternative polynomial fitting functions, with the aim of achieving a more accurate description of the data and, in turn, gaining deeper insight into the nature of the solute–solvent interactions in the studied systems.

## 3 Results and Discussion

### 3.1 Stability Evaluation by UV–Vis Spectroscopy

The chemical stability of the four polyphenols studied in all solvents was evaluated by comparing the UV–vis spectra in dissolution with time. Figure 1 shows, as an example, the UV–vis absorption graphs of *p*-coumaric acid in ethanol measured over 72 h, whereas the other spectra can be found in the Supporting Information (Figure S3). As for *trans*-polydatin, quercetin, and *trans*-resveratrol in methanol and ethyl acetate, chemical stability was already evaluated in a previous work.^42^

**Figure 1:**
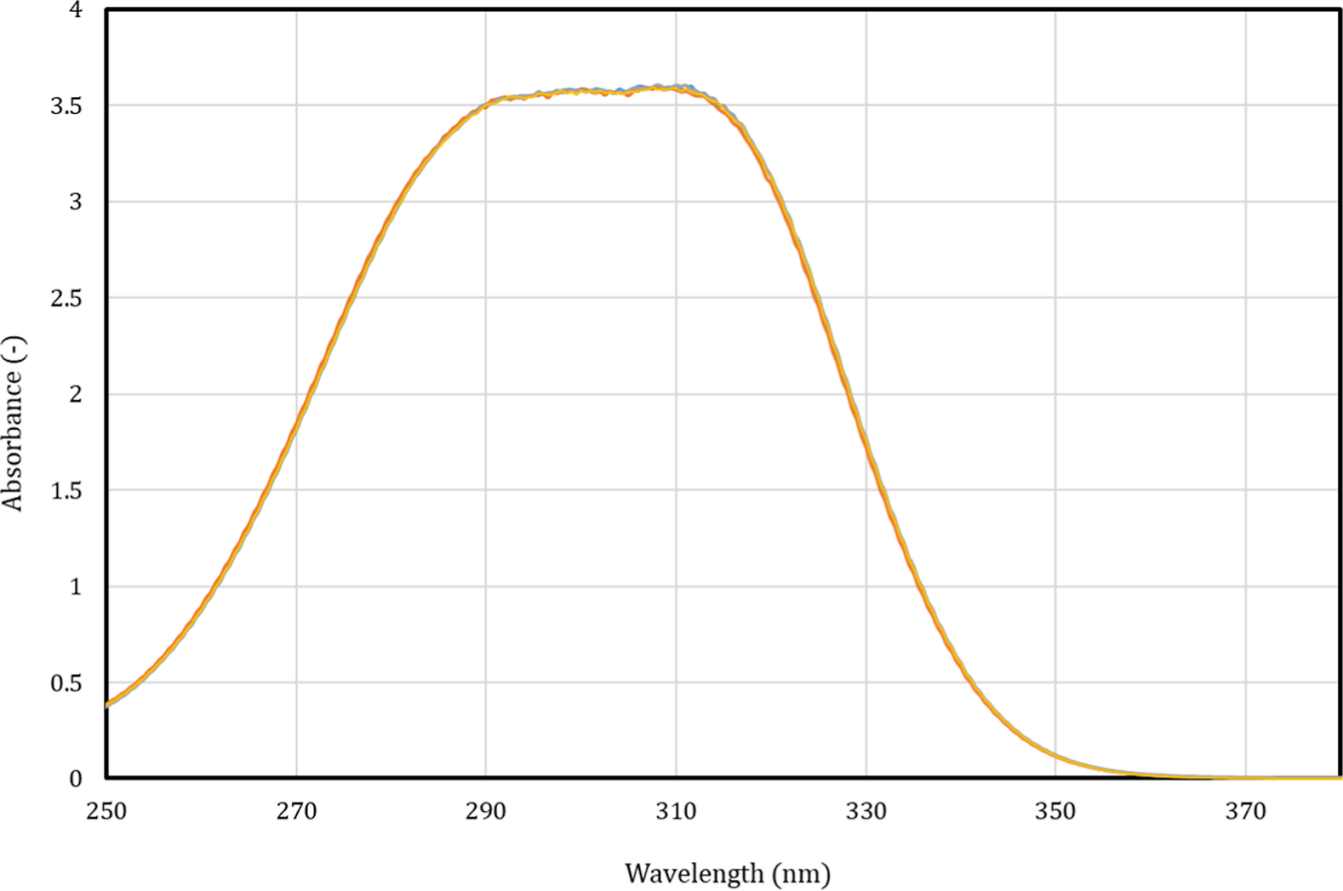
Ultraviolet spectra absorption graph of *p*-coumaric acid in ethanol, measured (—) 0 h, (—) 24 h, (—) 48 h, and (—) 72 h after dissolution.

As observed, all the absorption graphs are overlapped for each polyphenol in a given solvent (Δ*A* < 2% at maximum wavelength), which implies no significant variation with time. Thus, it can be concluded that all compounds are chemically stable in the three solvents within the studied time range.

### 3.2 Solubility Determination

#### 3.2.1 Pure Solvents

Equilibrium solubility of *trans*-polydatin, *p*-coumaric acid, quercetin, and *trans*-resveratrol in pure ethanol, and that of *p*-coumaric acid in pure methanol and ethyl acetate was measured at 298.2 K and 0.1 MPa. Experimental results, expressed as the mole fraction of solute, *x*, are displayed in Table 2, including previously measured solubilities.

**Table 2:** Experimental mole fraction solubility (*x*) of *trans*-polydatin, *p*-coumaric Acid, quercetin, and *trans*-resveratrol in methanol, ethanol, and ethyl acetate at 298.2 K and 0.1 MPa^a^

	*trans*-polydatin	*p*-coumaric acid	quercetin	*trans*-resveratrol
*x*·10^5^/mol fraction				
methanol	859.7 ^42^	2754	143.8 ^42^	1890 ^42^
ethanol	271.5	3352	601.9	2583
ethyl acetate	6.732 ^42^	968.5	223.0 ^42^ ^42^	895.0 ^42^

a The standard uncertainty of temperature is u­(*T*) = 0.1 K, and that of pressure is u­(*P*) = 1 kPa. Standard uncertainty for all mole fraction solubility values is u­(*x*) = 0.0005.

Higher solubilities were achieved in ethanol than in methanol for all polyphenols except *trans*-polydatin, whose solvation may be favored by the small size of methanol since it is a larger polyphenolic molecule. Conversely, *p*-coumaric acid, quercetin, and *trans*-resveratrol are more soluble in ethanol due to weaker solvent–solvent interactions. The highest solubility values are reached for *p*-coumaric acid in ethanol, probably because it has one less benzene ring than *trans*-resveratrol, thus enhancing the polarity and promoting solute–solvent interactions. On the other hand, all polyphenols studied present the lowest solubility in ethyl acetate, probably due to the absence of hydroxyl groups and longer carbonic chain. The only exception to this statement is the case of quercetin, which was already reported to exhibit a similar solubility in methanol and ethyl acetate.^42^

Table 3 collects experimental values found in the literature for comparison, along with some of those measured in this work. Comparison regarding *trans*-polydatin, quercetin, and *trans*-resveratrol in methanol and ethyl acetate was performed previously.^42^ The absolute relative deviation (ARD) is calculated as expressed in eq 5

5 $$ARD=\left|\frac{\left(x-x_{ref}\right)}{x_{ref}}\right|100$$

where *x* is the mole fraction solubility calculated in this work and *x*_ref_ is a reference mole fraction solubility value, taken in this case from the literature.

**Table 3:** Experimental mole raction folubilities btained in this study^a^, along with referenced iterature values, all measured at 298.2 K and 0.1 MPa

*x*·10^5^/mol fraction			
polyphenol/solvent	methanol	ethanol	ethyl acetate
*trans*-polydatin	859.7 ^42^ ^b^	271.5 (this work)	6.732 ^42^ ^b^
*p*-coumaric acid	2754 (this work)	3352 (this work)	968.5 (this work)
	3950 ^45^	4560 ^45^	1280 ^45^
	4269 ^46^	5006 ^46^	1046 ^46^
		4612 ^47^	1293 ^47^
quercetin	143.8 ^42^ ^b^	601.9 (this work)	223.0 ^42^ ^b^
		153.0 ^49^	
		250.5 ^48^	
*trans*-resveratrol	1890 ^42^ ^b^	2583 (this work)	895.0 ^42^ ^b^
		1690 ^51^	
		1660 ^50^	
		2480 ^52^	

a The standard uncertainty of temperature is u­(*T*) = 0.1 K, and that of pressure is u­(*P*) = 1 kPa. Standard uncertainty for all mole fraction solubility values is u­(*x*) = 0.0005.

b The literature comparison of these systems is covered in a previous work.^42^

As it can be extracted from Table 3, Ji et al.,^45^ Vilas-Boas et al.,^46^ and Noubigh et al.^47^ obtained the same solubility order as we did in this work for *p*-coumaric acid: it is more soluble in ethanol than in methanol, and lower in ethyl acetate than in methanol. Although their values are higher than ours, all methodologies applied differ to a certain extent from our procedure, since some of them quantified gravimetrically without drying and others filtered instead of centrifuging, with the consequent interference that this may entail in the quantification. As for quercetin in ethanol, large deviations are obtained with respect to the data measured by Malwade et al.^48^ and Razmara et al .,^49^ being more than two and almost four times lower than ours, respectively. However, quercetin hydrate was used in both studies instead of pure quercetin, and no drying was performed before experimentation. The measured solubility of *trans*-resveratrol in ethanol differs up to 55.6% and 52.8% with that obtained by Ghazwani et al.^50^ and Sun et al.,^51^ respectively, whereas it largely agrees with the value provided by Ha et al.,^52^ deviating in less than 4.2%, revealing a considerable discrepancy among different literature studies. Finally, no data were found in the literature on experimental solubility of *trans*-polydatin in ethanol.

#### 3.2.2 Binary Solvent Mixtures

The experimental mole fraction solubility of *trans*-polydatin (x_p_), *p*-coumaric acid (x_c_), quercetin (x_q_), and *trans*-resveratrol (x_r_) in the binary system ethanol + ethyl acetate, and that of *p*-coumaric acid and *trans*-resveratrol in the binary mixture methanol + ethyl acetate were determined at 298.2 K and 0.1 MPa for the whole range of solvent composition, in order to study the effect of the acetate solvent. Results are plotted in Figure 2 and reported in Table S3.

**Figure 2:**
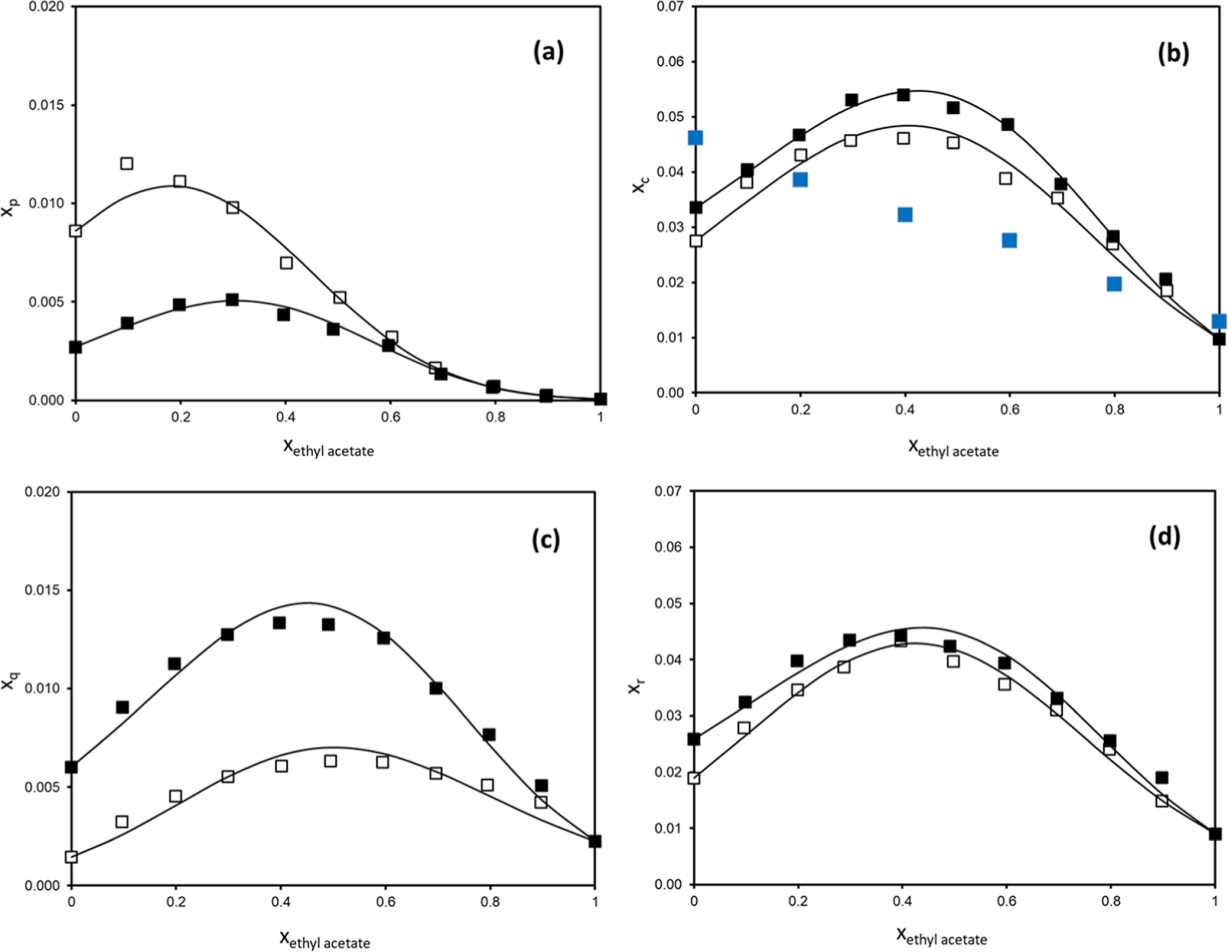
Experimental mole fraction solubility of (a) *trans*-polydatin (*x*_p_), (b) *p*-coumaric acid (*x*_c_), (c) quercetin (*x*_q_), and (d) *trans*-resveratrol (*x*_r_) in methanol + ethyl acetate (□) and ethanol + ethyl acetate (■), as a function of the mole fraction of ethyl acetate in the binary solvent mixture (*x*_ethylacetate_). Experimental solubility data of *trans*-polydatin and quercetin in methanol + ethyl acetate binary solvent mixture were extracted from a previous work.^42^ Solubility of *p*-coumaric acid in ethanol + ethyl acetate from Noubigh et al.^52^ (■) is displayed for comparison. Solid lines represent the fitting by CNIBS/R-K model for each system.

As observed in Figure 2, all polyphenols achieved a maximum solubility at a specific concentration of the binary solvent in all systems studied, which reveals a synergistic effect between ethyl acetate and both alcohols. The greatest solubility enhancement was observed for *trans*-resveratrol, with a 129% increase in methanol + ethyl acetate at 0.4 mole fraction of ethyl acetate, compared to the solubility in pure methanol, closely followed by the 121% maximum enhancement of quercetin solubility at 0.4 mole fraction of ester solvent in ethanol + ethyl acetate with respect to pure ethanol. *trans*-Polydatin increased its solubility by 88.5% at 0.3 mole fraction of ethyl acetate in the ethanol + ethyl acetate mixture, while its maximum in the methanol + ethyl acetate binary solvent mixture was previously demonstrated at 0.1 mole fraction of ethyl acetate,^42^ which denotes a significant shift. Solubility enhancements for *p*-coumaric acid are quite similar in both ethanol + ethyl acetate and methanol + ethyl acetate solvent mixtures, with increases at 0.4 mole fraction of ethyl acetate of approximately 61.0% and 67.3%, respectively, compared to solubility values in pure alcohols.

It must be highlighted that all polyphenols studied attain higher maximum solubilities in ethanol + ethyl acetate than in methanol + ethyl acetate, with *trans*-polydatin being the only exception, which is also more soluble in pure methanol than ethanol. This way, it appears that the differences in solubility maxima could be predicted as a function of the solubilities of polyphenols in pure solvents for the studied systems: quercetin, *p*-coumaric acid, and *trans*-resveratrol show a higher solubility in an ethanol + ethyl acetate solvent mixture compared to that containing methanol; conversely, *trans*-polydatin is more soluble in the latter for the whole composition range. These results suggest that it would be possible to expect in which binary system these compounds would present the highest solubility only based on solubility data in pure solvents. For instance, *trans*-polydatin is the only polyphenol of the four studied that is more soluble in methanol than in ethanol and also presents a higher solubility in the binary solvent mixture that contains methanol.

Furthermore, the mole fraction at which the maximum solubility is achieved in each case also seems to depend on the solubility of a given polyphenol in the pure solvents that conform to the binary mixtures. In Figure 2, it can be observed that the solubility maximum of *trans*-polydatin and quercetin in both binary systems is achieved at different compositions; in the case of *trans*-polydatin, it is reached at 0.3 mole fraction of ethyl acetate in the binary mixture containing methanol, whereas in that formed by ethanol and ethyl acetate, the solubility maximum is attained at 0.1 mole fraction of the ester solvent. As for quercetin, this occurs at 0.5 and 0.4 mole fractions of ethyl acetate, respectively. This could be explained by the fact that the solubility of these two compounds in one of the two alcohols is 3 or 4 times higher than in the other: *trans*-polydatin is 3 times more soluble in methanol than in ethanol, leading to a solubility maximum at a lower ethyl acetate concentration in the methanolic mixture compared to the ethanolic mixture. This same phenomenon occurs with quercetin.

As for *trans*-resveratrol and *p*-coumaric acid, however, solubility maxima are observed for the same composition in both studied binary mixtures since the solubility of both polyphenols in ethanol and methanol is quite similar.

This premise—that the maximum solubility can be predicted from the solubility data of the polyphenol in the pure components—is confirmed in the eight systems studied, where it is observed that the smaller the difference between the solubility values of the polyphenol in the pure compounds forming the binary mixtures, the more the solubility maxima of each polyphenol in the binary mixtures shift toward the pure component with lower solubility.

Contrary to solubility in pure solvents, literature data were found only for *p*-coumaric acid in ethanol + ethyl acetate. Our results for that ternary system disagree to a great extent from those provided by Noubigh et al.,^47^ mainly because they concluded that solubility of *p*-coumaric acid in ethanol + ethyl acetate behave linearly with concentration, while our experience revealed a solubility enhancement. The solubility determination method applied by the authors differs significantly from our methodology, since they did not dry the polyphenols and used a gravimetric process for equilibrium and determination, while UV–vis was applied in this work for quantification.

### 3.3 PXRD Analysis

PXR diffractograms of the four raw polyphenols, together with their recrystallized samples, were obtained from saturated solutions in methanol, ethanol, and ethyl acetate. Results for *trans*-polydatin, quercetin, and *trans*-resveratrol after solubilization in ethanol are displayed in Figures S4–S6 of the Supporting Information (crystallization behavior in methanol and ethyl acetate was discussed in a previous work), whereas diffractograms of *p*-coumaric acid are presented in Figure 3. The similarity observed in both diffraction peak positions and full widths at half-maximum (FWHM) across all samples provides strong evidence that none of the studied polyphenols exhibited polymorphic behavior under the recrystallization conditions in all pure solvents tested. However, it should be highlighted that peak intensity of the diffractograms of *trans*-resveratrol and quercetin after solubilization in ethanol is lower than that of the raw material, which can be due to the reduced mass employed for the PXRD tests of the former cases.

**Figure 3:**
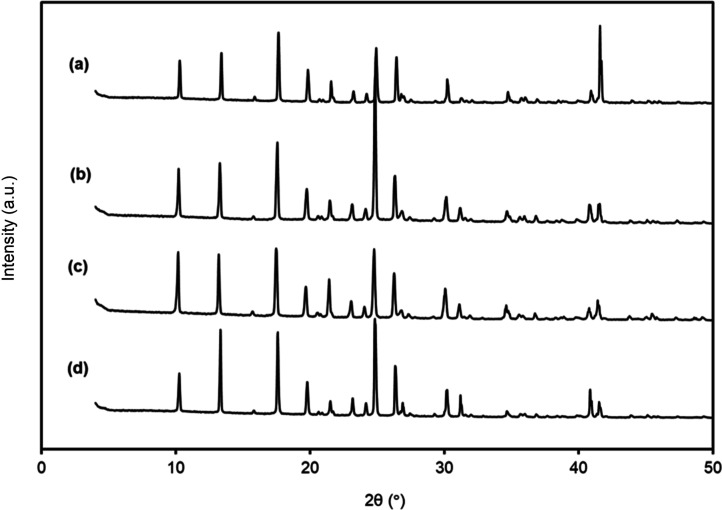
PXRD profiles of *p*-coumaric acid: (a) raw material, and recrystallized after solubilization in (b) methanol, (c) ethanol, and (d) ethyl acetate.

This consistency in the diffraction profiles indicates that the crystalline structure of all polyphenols was preserved after solubilization in methanol, ethanol, and ethyl acetate.

### 3.4 Solubility Modeling

#### 3.4.1 Abraham Solvation Model

The solubility of *p*-coumaric acid, quercetin, and *trans*-resveratrol in methanol, ethanol, and ethyl acetate at 298.2 K and 0.1 MPa was predicted using the Abraham solvation model in order to assess the capacity of the LFER framework to reproduce the experimental solubility of polyphenols in pure organic solvents by using molecular descriptors.

Figure 4 shows the deviations of the predicted results using the Abraham solvation model from the experimental solubility data obtained in this work. Quite accurate estimations were found for *trans*-resveratrol in ethanol and *p*-coumaric acid in ethyl acetate, with ARDs of 8.64% and 8.46%, respectively, being the lowest ones. In fact, *trans*-resveratrol and *p*-coumaric acid achieved an average ARD of less than 18.5%, whereas quercetin attained a value of 30.6%. This remarkable difference can be attributed to the size and hydrogen bond behavior of quercetin, which is much larger and has more hydrogen bond donors and acceptors than *trans*-resveratrol and *p*-coumaric acid, two factors that might compromise the correlation to a certain extent. As far as solvents are concerned, it was observed that the more polar the solvent the higher the average ARD, being 15.7%, 16.5%, and 29.4% for solubility in ethyl acetate, ethanol, and methanol, respectively, which highlights the impact of polarity in the model precision. In fact, this trend aligns with the dielectric constant of the solvents, standing for 6.1, 25.3, and 33 for ethyl acetate, ethanol, and methanol,^58^ which reinforces the influence of the permanent dipoles present in alcohols in the solubility, and consequently in the model performance. Overall, though, an average ARD of 20.5% indicates that the Abraham solvation model can generally provide good estimations of the solubility of the three polyphenols in the solvents studied.

**Figure 4:**
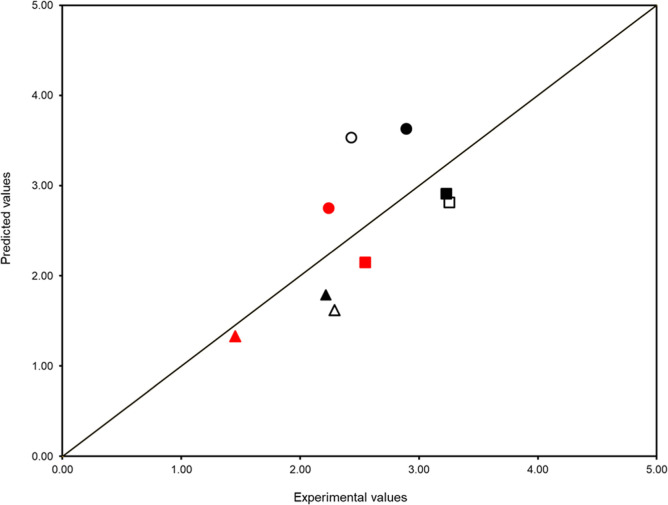
Comparison between the experimental and calculated $\log\left(\frac{C_{s}}{C_{w}}\right)$ values with the Abraham solvation model, at 298.2 K and 0.1 MPa, of *p*-coumaric acid (Δ), quercetin (○), and *trans*-resveratrol (■) in pure methanol (□), ethanol (■), and ethyl acetate (■).

Additionally, contributions of single descriptors to the solubility of polyphenols were also analyzed. Results revealed that polarizability (*S* and *s*), and the McGowan molecular volume (*V* and *v*) descriptors, were the largest contributors to solubility partition. The polarizability term represented between 24.0 and 39.6% of the total, whereas the molecular volume contributed between 15.9% and 45.8%. In fact, it was observed that the predominant Abraham descriptor of *trans*-resveratrol and quercetin in methanol and ethanol was the polarizability, averagely standing for 26.2% of total solubility, which is in accordance with the high polarity of both alcohols with respect to ethyl acetate. Conversely, the McGowan molecular volume seems to be the main contributor to solubility of the three polyphenols in ethyl acetate, representing the 41.2%, 39.0%, and 45.8% for *trans*-resveratrol, quercetin, and *p*-coumaric acid, respectively.

#### 3.4.2 Solubility Excess

Deviation from the ideal solubility was calculated for *trans*-polydatin, *p*-coumaric acid, quercetin, and *trans*-resveratrol in binary solvent mixtures at 298.2 K and 0.1 MPa. Excess values are depicted in Figure 5, from which it can be noted that all polyphenols exhibit positive deviations in the whole mole fraction range of both binary solvent mixtures. This behavior is in accordance with the overall solubility enhancement observed in the solubility profiles; nevertheless, there is no clear correspondence between the binary solvent composition at which the highest excess and solubility maxima are achieved (Table S3). In contrast, a certain trend in the excess solubility can be inferred based on the size of the solute, such that the higher the molecular weight of the polyphenol the greater the deviation from ideality: *trans*-polydatin > quercetin > *trans*-resveratrol > *p*-coumaric acid. This tendency is more pronounced in methanol + ethyl acetate, while in ethanol + ethyl acetate, the excess solubility of *p*-coumaric acid and *trans*-resveratrol are quite similar regardless of the solvent mixture composition.

**Figure 5:**
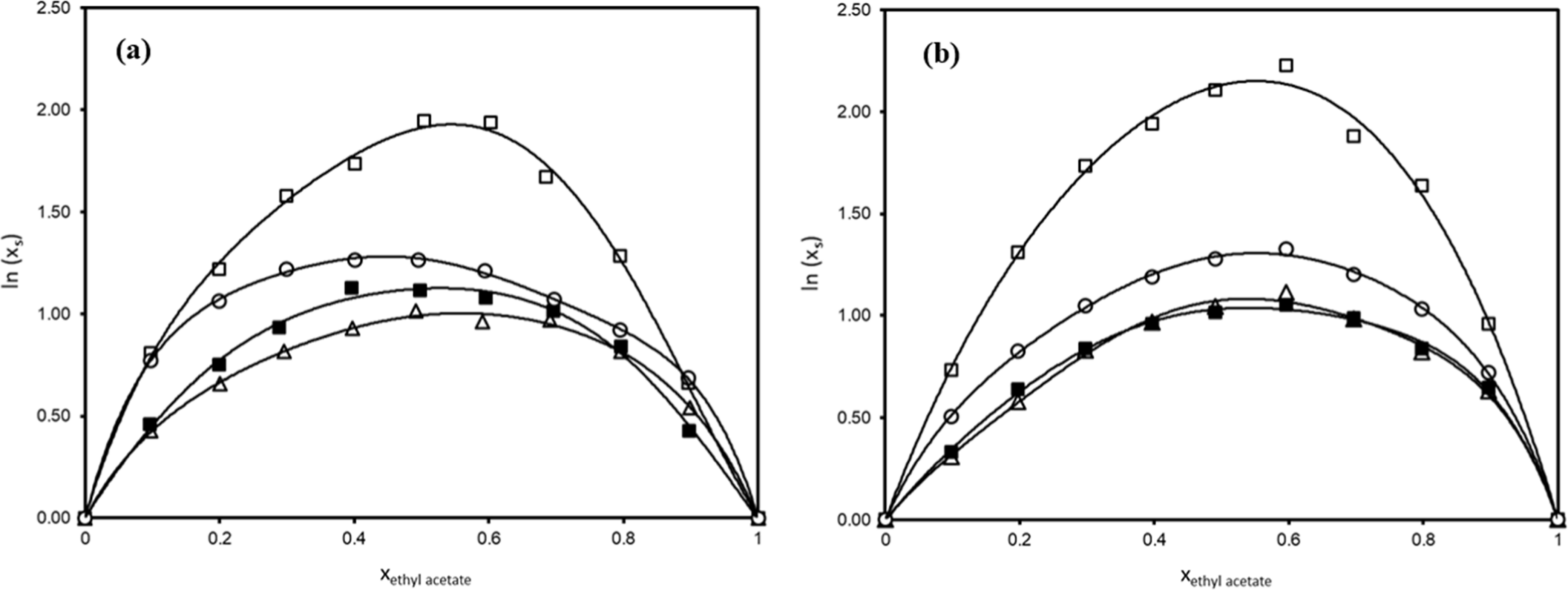
Solubility excess values of *trans*-polydatin (□), *p*-coumaric acid (Δ), quercetin (○), and *trans*-resveratrol (■) in (a) methanol + ethyl acetate and (b) ethanol + ethyl acetate, as a function of the mole fraction of ethyl acetate in the binary solvent mixture (*x*_ethylacetate_). Solid lines are displayed for better visualization.

#### 3.4.3 CNIBS/R–K Solubility Model

The calculated parameters, coefficients of determination (*R* ^2^), and relative mean square deviations (RMSD) of results from the correlation applying the CNIBS/R–K equation are collected in Table 4, while fitting is depicted in Figure 2. It can be inferred that the proposed model can correlate the experimental solubility data with high accuracy using only 2 model constants, especially for polyphenols in ethanol + ethyl acetate solvent mixture, achieving *R* ^2^ values between 0.984 and 0.994. On the contrary, a slightly less precise prediction was attained for quercetin in methanol + ethyl acetate, with *R* ^2^ = 0.943.^42^ In order to avoid overfitting and/or underfitting, other approaches were proposed for the eight systems using 1, 3, and 4 model constants, achieving worse results and thus weaker descriptions of the experimental solubility values.

**Table 4:** Model Parameters, *R* ^2^, and RMSD^a^ Values of *trans*-Polydatin, *p*-Coumaric Acid, Quercetin and *trans*-Resveratrol in Methanol + Ethyl Acetate and Ethanol + Ethyl Acetate Solvent Mixtures, Obtained Using the CNIBS/R–K Solubility Model

polyphenol	*S*_0_ ^a^	*S*_1_ ^a^	*R* ^2^	RMSD^b^
methanol + ethyl acetate				
*trans*-polydatin	7.7174	–0.3212	0.985	0.059
*p*-coumaric acid	4.2020	–0.5472	0.978	0.041
quercetin	5.5012	0.8215	0.943	0.108
*trans*-resveratrol	4.6619	–0.0721	0.990	0.023
ethanol + ethyl acetate				
*trans*-polydatin	8.7259	–1.2030	0.990	0.060
*p*-coumaric acid	4.3500	–1.2294	0.994	0.044
quercetin	5.4090	–0.9581	0.984	0.058
*trans*-resveratrol	4.3347	–1.0773	0.984	0.044

a Values are reported with 95% confidence intervals (coverage factor *k* = 2).

b Calculated as $RMSD={\left[\frac{1}{N}\sum_{i=1}^{N}{\left(x_{cal}-x\right)}^{2}\right]}^{1/2}$ , N being the number of experimental groups and *x*_cal_ the mole fraction solubility calculated with the CNIBS/R–K model.

#### 3.4.4 Kamlet–Taft Solvatochromic Parameters

To adjust the solubility of the solvent with the solvent composition, the solvatochromic parameters of the binary solvent mixtures must be known. The parameters α, β, π* and the Hildebrand solubility parameter δ_H_^2^ for the binary systems ethanol + ethyl acetate and methanol + ethyl acetate are available in the literature,^59^ and are collected in Table S4 and Figure S7. It can be observed that the polarizability parameter remains constant regardless of composition, while both α and β decrease as the concentration of ethyl acetate increases. This indicates that an increase in the ethyl acetate composition in the mixture reduces both the acidity and basicity of the solvent. Similarly, the δ_H_^2^ parameter decreases as the ethyl acetate concentration rises.

In this study, it was considered that solvatochromic parameters α, β, and π* contribute positively to solubility, as they promote dissolution, whereas the contribution of the Hildebrand solubility parameter was evaluated as negative, since additional energy must be supplied to create molecular gaps. These opposing contributions are consistent with the observed solubility data since all polyphenols studied exhibit a maximum solubility at a specific composition of the binary solvent mixture (see Figure 2).

Given these considerations, a linear equation such as the one proposed by KAT in the KAT-LSER model does not adequately describe the solubility data for our systems. Therefore, in this work, we attempted to fit the data using nonlinear equations. The equations employed were of the type of eq 6.

6 $$x=C_{o}+C_{1}\cdot \pi^{*}+C_{2}{\cdot \left(\pi^{*}\right)}^{2}+C_{3}\cdot \beta +C_{4}{\cdot \beta }^{2}+C_{5}\cdot \alpha +C_{6}{\cdot \alpha }^{2}+C_{7}\cdot \left(\delta_{H}^{2}/1000\right)+C_{8}\cdot {\left(\delta_{H}^{2}/1000\right)}^{2}$$

in which solubility exhibits a second-order dependence on each of the four solvatochromic parameters. Initially, since π* appeared unlikely to exert a significant effect on solubility, all possible equations containing this parameter were tested, confirming—as expected—that it had no influence. Consequently, π* was excluded as a solubility-determining term and the three remaining parameters, α, β, and δ_H_^2^ were then combined into a set of equations as follows

7 $$x=C_{o}+C_{1}\cdot \beta +C_{2}{\cdot \beta }^{2}+C_{3}\cdot \alpha +C_{4}{\cdot \alpha }^{2}+C_{5}\cdot \left(\delta_{H}^{2}/1000\right)+C_{6}\cdot {\left(\delta_{H}^{2}/1000\right)}^{2}$$

which were iteratively tested and refined. Special care was taken when correlating the data to avoid any expected overfitting due to the high number of initial coefficients in the iterations: although beginning with seven parameters indeed implies a higher risk of overfitting, this initial choice was justified by the need to account for three potential solvatochromic contributions (α, β, and δ_H_^2^) and to properly describe the enhanced-solubility behavior. Nonetheless, each case was evaluated individually using appropriate statistical criteria that helped to detect overfitted results. Consequently, this process revealed that certain parameters did not exert a significant influence, leading to progressive reductions in the number of coefficients, from the original seven down to six, five, four, and finally three coefficients. Examination of the results from all tested equations led to the conclusion that the equations providing the best fit for the solubility data of the systems are those presented in Tables 5 and 6, which includes only 3 or 4 parameters. Furthermore, Figure 6 shows the solubility data of the four polyphenols analyzed as a function of the ethyl acetate fraction in the binary mixtures. The experimental results are compared to the values calculated from the best fit obtained using solvatochromic parameters. In both cases, a good correlation between the experimental and fitted values is observed, confirming the validity of the applied model to describe the solubility behavior in these systems.in Table 5, the coefficients *C*_1_ and *C*_2_ in the equations *x* = *C*_o_ + *C*_2_·β^2^+ *C*_6_·(δ_H_^2^)^2^ and *x* = *C*_o_ + *C*_1_·β+ *C*_6_·(δ_H_^2^)^2^ indicate that the acidity of the hydrogen bonds between the solute and the solvent favors dissolution. Similarly, the coefficient C_3_ in the equation *x* = *C*_o_ + *C*_3_·α + *C*_6_·(δ_H_^2^)^2^ reveals that the basicity of the hydrogen bonds between the solute and solvent also promotes solubility. In both expressions, the role of the Hildebrand solubility parameter is further emphasized by the coefficient *C*_6_.

**Table 5:** Fitting Coefficients and RMSD Values Corresponding to the Optimal Correlation Equation for the Solubility of the Four Polyphenols with the Solvatochromic Parameters in the Ethanol + Ethyl Acetate Binary Mixture

*x* = *C*_o_ + *C*_2_·β^2^ + *C*_6_·(δ_H_^2^)^2^	*C*_0_	*C*_1_	*C*_2_	*C*_3_	*C*_4_	*C*_5_	*C*_6_	RMSD
*p*-coumaric	–0.328	–	4.483	–	–	–	–3.842	0.189
*trans*-resveratrol	–0.265	–	3.664	–	–	–	–3.148	0.087
quercetin	–0.092	–	1.261	–	–	–	–1.088	0.035
*trans*-polydatin	–0.033	–	0.424	–	–	–	–0.358	0.060
*x* = *C*_o_ + *C*_1_·β+ *C*_6_·(δ_H_^2^)^2^								
*trans*-polydatin	–0.049	0.136	–	–	–	–	–0.089	0.049
*x* = *C*_o_ + *C*_3_·α + *C*_6_·(δ_H_^2^)^2^								
*trans*-polydatin	–0.014	–	–	0.052	–	–	–0.098	0.042

**Table 6:** Fitting Coefficients and RMSD Values Corresponding to the Optimal Correlation Equation for the Solubility of the Four Polyphenols with the Solvatochromic Parameters in the Methanol + Ethyl Acetate Binary Mixture

*x* = *C*_o_ + *C*_3_·α + *C*_4_·α^2^ + *C*_6_·(δ_H_^2^/1000)^2^	*C*_0_	*C*_1_	*C*_2_	*C*_3_	*C*_4_	*C*_5_	C_6_	RMSD
*p*-coumaric	1.172	–	–	3.231	1.863	–	–7.891	0.127
*trans*-resveratrol	0.767	–	–	2.123	1.184	–	–5.121	0.211
quercetin	0.164	–	–	0.439	0.261	–	–1.089	0.011
*x* = *C*_o_ + *C*_3_·α + *C*_6_·(δ_H_^2^/1000)^2^								
*trans*-polydatin	0.010	–	–	0.065	–	–	–0.083	0.111

**Figure 6:**
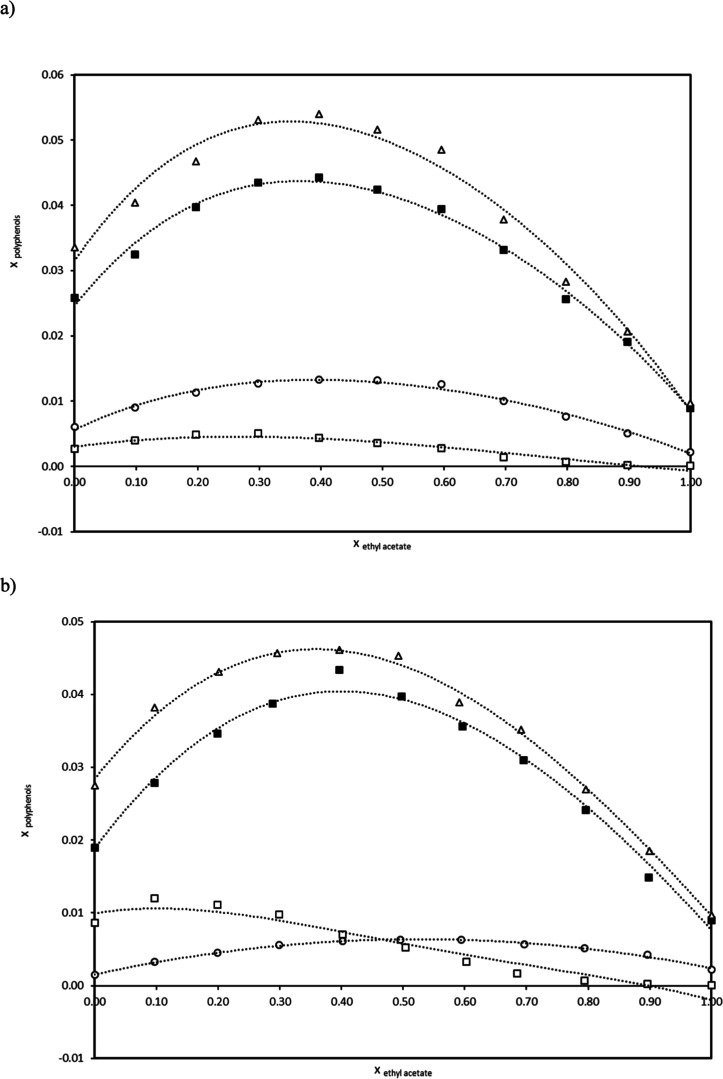
Experimental mole fraction solubility of *trans*-polydatin (□), *p*-coumaric acid (Δ), quercetin (○), and *trans*-resveratrol (■) in (a) ethanol + ethyl acetate and (b) methanol + ethyl acetate, as a function of the mole fraction of ethyl acetate in the binary solvent mixture (*x*_ethylacetate_). Solid lines represent the best fit obtained using solvatochromic parameters for each system.

As shown in Table 5, the equation that best fits the solubility data of *p*-coumaric acid, *trans*-resveratrol, and quercetin in the binary mixture ethanol + ethyl acetate is the one in which the most influential solvatochromic parameter on solubility is β, along with the Hildebrand solubility parameter, δ_H_^2^. In contrast, for *trans*-polydatin, the influence of parameter β decreases in favor of parameter α, although the change in the quality of the fit is not particularly significant.

Table 6 indicates that for the binary mixtures with methanol, the best fit of the solubility data for the four polyphenols was obtained using the α parameter, together with the Hildebrand solubility parameter.

The fact that three distinct equations effectively describe the solubility data—one predominantly influenced by the α parameter, another by the β parameter, and the third incorporating both—can be explained by analyzing the molecular structures of the solutes. All six polyphenols possess functional groups that enable them to act as both hydrogen bond donors and acceptors, reflecting the complexity of the multiple interactions that govern their solubility in the solvent mixtures studied.

The relevance of the Hildebrand solubility parameter in these systems is likely attributable to the relatively large size of the solute molecules, which requires a high cohesive energy for solvation. Further insights can be drawn from Table S4 and Figure S7: Table S4 shows that pure ethyl acetate has a α parameter value of zero, whereas for alcohols, this value approaches to 1; additionally, the β parameter of pure ethyl acetate is consistently lower than that of alcohols. Figure 6 demonstrates that the solubility of polyphenols reaches its maximum at intermediate ethyl acetate concentrations in all of the studied systems. This trend suggests that enhanced solute dissolution is achieved by partially reducing both the acidity and basicity of the solvents.

Furthermore, the observation that solubility maxima also occur at intermediate values of α and β reinforces the idea that these parameters play a fundamental role in dictating the dissolution behavior of the six polyphenols in these binary solvent systems.

## 4 Conclusions

The solubility of *trans*-polydatin, *p*-coumaric acid, quercetin, and *trans*-resveratrol in pure ethanol and the solubility of *p*-coumaric acid in pure methanol and ethyl acetate was determined at 298.2 K and 0.1 MPa by the equilibrium method. Highest values were attained in ethanol, except for *trans*-polydatin, which is preferentially solvated by methanol. Unlike alcohols, ethyl acetate achieved the lowest solubilities for all compounds tested. PXRD analysis confirmed that no structural changes occurred in the studied polyphenols after recrystallization from the solvents tested, confirming that the solubilization did not alter their crystalline nature.

The same method was applied to measure the solubility of those polyphenols in binary solvent mixtures containing ethanol + ethyl acetate and that of *trans*-resveratrol and *p*-coumaric acid in the binary solvent mixture composed of methanol + ethyl acetate, covering the whole molar fraction range. All polyphenols exhibited solubility maxima in all binary solvent mixtures, due to the synergistic behavior between alcohols and ethyl acetate. The positive excess solubility values further confirmed this synergistic effect, highlighting the nonideal mixing behavior of the solvents. In addition, solubility of polyphenols in pure solvents was found to successfully predict the solvent system and composition at which maximum solubility is achieved. For instance, polyphenols more soluble in ethanol than in methanol showed higher maxima in ethanol + ethyl acetate binary mixtures, while *trans*-polydatin, more soluble in methanol, revealed the opposite trend.

Experimental solubility data of the four polyphenols in binary mixtures were successfully correlated by using the CNIBS/R-K equation, achieving high coefficients of determination for all systems studied. For its part, Abraham solvation model demonstrated strong robustness in predicting solubility of polyphenols in pure solvents, and a reliable description of solubility was obtained using the solvatochromic parameters of hydrogen-bond acidity and basicity, α and β, together with the Hildebrand solubility parameter, δ_H_^2^, while it was observed that the polarity parameter, π*, does not influence the systems under study.

Overall, the results of this study underscore the importance of experimental solubility measurements as a practical tool for solvent selection toward recovery of bioactive compounds, as well as the relevance of the integration of predicting modeling approaches with experimental data for rationally designing efficient extraction systems, which is particularly significant for pharmaceutical and cosmetic industries.

## Supplementary Material



## Notes

Chemical structure, calibration curves, for solubility determination, ultraviolet spectra absorption graphs, and PXR diffractograms of polyphenols; solvatochromic parameters of binary solvent mixtures and Abraham descriptors of solutes and tested solvents; and mole fraction solubility of polyphenols in binary solvent mixtures at 298.2 K and 0.1 MPa (PDF)
