# Supplementary material for: Experimental and Modeling Assessment of Polyphenol Solubility in Alcohol + Ethyl Acetate Mixtures for Extraction Applications
Source: ACS Omega. 2026 Feb 26;11(9):14774–86. doi: 10.1021/acsomega.5c10953 (PMC12980224; doi:10.1021/acsomega.5c10953)
Supplement: Supplementary file 1 [file ao5c10953_si_001.pdf]

# *Experimental and Modeling Assessment of Polyphenol Solubility in Alcohol + Ethyl Acetate Mixtures for Extraction Applications*

Iván Montenegro, Begoña González, Ángeles Domínguez, Elena Gómez*

FEQx lab, Department of Chemical Engineering, University of Vigo, Spain

* Corresponding author

E-mail address: elenagc@uvigo.es

## Supplementary material

- **Figure S1.** Chemical structures of (a) *trans*-resveratrol, (b) *trans*-polydatin, (c) *p*-coumaric acid, and (d) quercetin.
- **Figure S2**. Calibration curves of (a) *trans*-polydatin, (b) *p*-coumaric acid, (c) quercetin, and (b) *trans*-resveratrol in methanol, measured via UV-VIS spectrophotometry and expressed as mass fraction of polyphenol against absorbance. Selected wavelengths for calibration were 305 nm for *trans*-polydatin and *trans*-resveratrol, 311 nm for *p*-coumaric acid, and 370 nm for quercetin.
- **Figure S3**. Ultraviolet spectra absorption graphs of (a) *trans*-polydatin, (b) *p*-coumaric acid, (c) quercetin, and (d) *trans*-resveratrol (1) methanol, (2) ethanol, and (3) ethyl acetate, measured (—) 0 h, (—) 24 h, (—) 48 h, and (—) 72 h after dissolution.
- **Figure S4.** PXRD profiles of *trans*-polydatin: (a) raw material, and (b) recrystallized after solubilization in ethanol.
- **Figure S5.** PXRD profiles of quercetin: (a) raw material, and (b) recrystallized after solubilization in ethanol.
- **Figure S6.** PXRD profiles of *trans*-resveratrol: (a) raw material, and (b) recrystallized after solubilization in ethanol.
- **Figure S7.** Solvatochromic parameters, (○) α, (●) β, (□) π*, and (△) Hildebrand solubility parameter, $\delta_H^2$, as a function of the mole fraction of the ethyl acetate ($x_{ethyl\ acetate}$) for the a) ethanol + ethyl acetate and b) methanol + ethyl acetate binary systems.
- **Table S1** Abraham descriptors of studied polyphenols, obtained from the UFZ-LSER database [1] and Vilas-Boas *et al.* [2][a].
- **Table S2** Abraham descriptors of tested solvents, obtained from the UFZ-LSER database [1].
- **Table S3**. Mole fraction solubility of *trans*-polydatin ($x_p$), *p*-coumaric ($x_c$), quercetin ($x_q$), and *trans*-resveratrol ($x_r$) in ethanol + ethyl acetate, and mole fraction solubility of *trans*-resveratrol ($x_r$) and *p*-coumaric ($x_c$) in methanol + ethyl acetate at 298.2 *K* and 0.1 MPa, all expressed as a function of the mole fraction of ethyl acetate ($x_{ethyl\ acetate}$) in the binary solvent mixtures[a].
- **Table S4.** Solvatochromic parameters, α, β and π*and Hildebrand solubility parameter, $\delta_H^2$, as a function of the mole fraction of the ethyl acetate ($x_{ethyl\ acetate}$) for the ethanol + ethyl acetate and methanol + ethyl acetate binary systems.

(a)

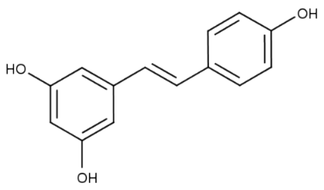


(b)

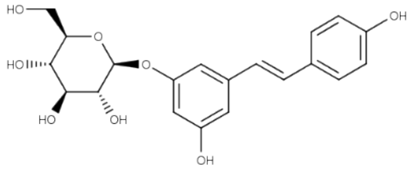


(c)

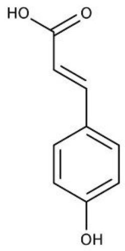


(d)

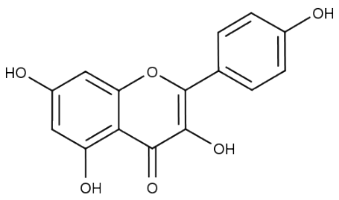


**Figure S1.** Chemical structures of (a) *trans*-resveratrol, (b) *trans*-polydatin, (c) *p*-coumaric acid, and (d) quercetin.

a)

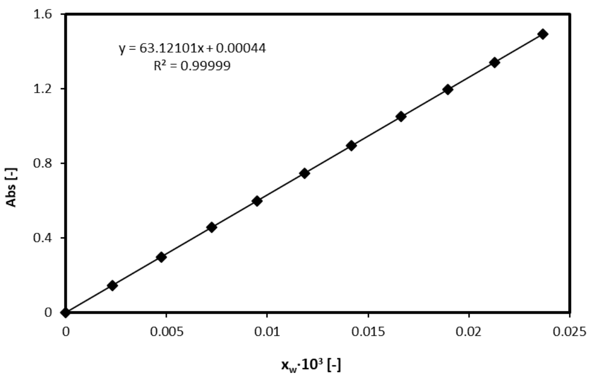


b)

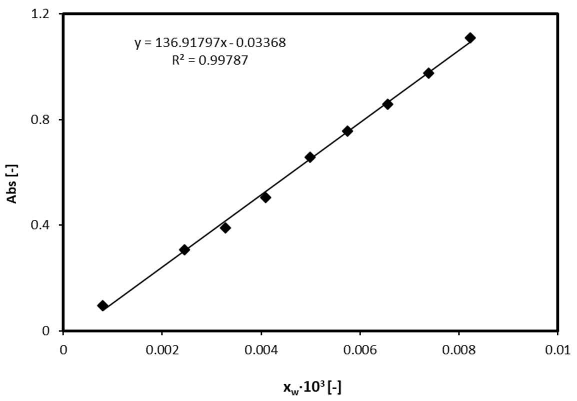


c)

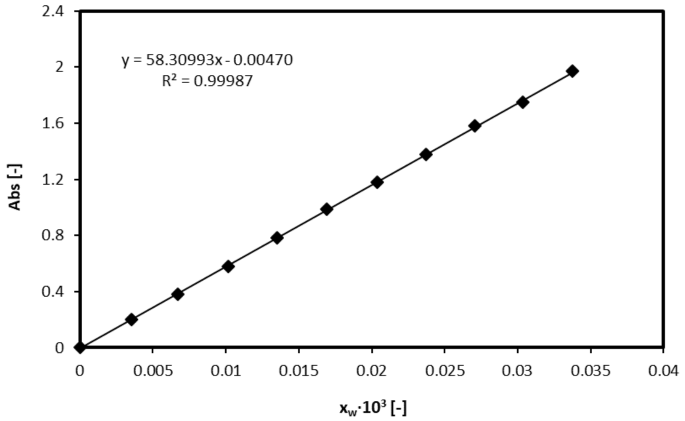


d)

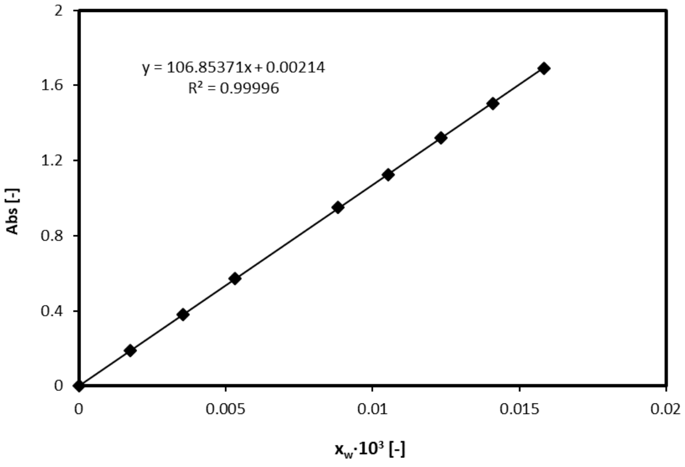


**Figure S2**. Calibration curves of (a) *trans*-polydatin, (b) *p*-coumaric acid, (c) quercetin, and (b) *trans*-resveratrol in methanol, measured via UV-VIS spectrophotometry and expressed as mass fraction of polyphenol against absorbance. Selected wavelengths for calibration were 305 nm for *trans*-polydatin and *trans*-resveratrol, 311 nm for *p*-coumaric acid, and 370 nm for quercetin.

a1)

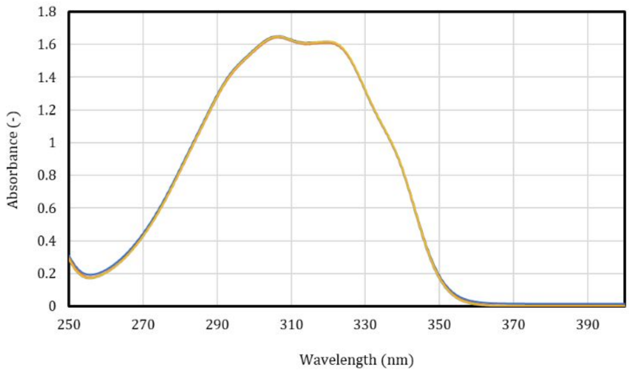

Absorbance (-)
Wavelength (nm)


a2)

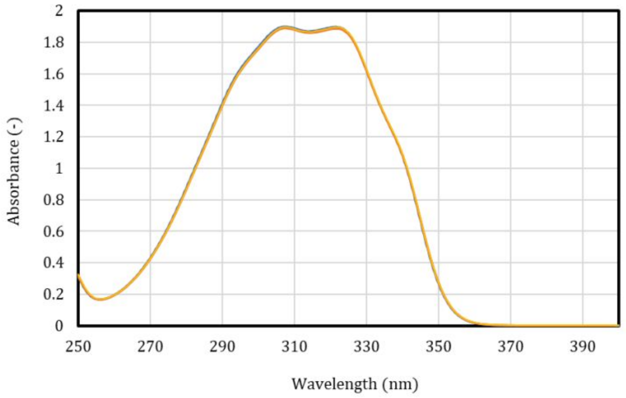

Absorbance (-)
Wavelength (nm)


a3)

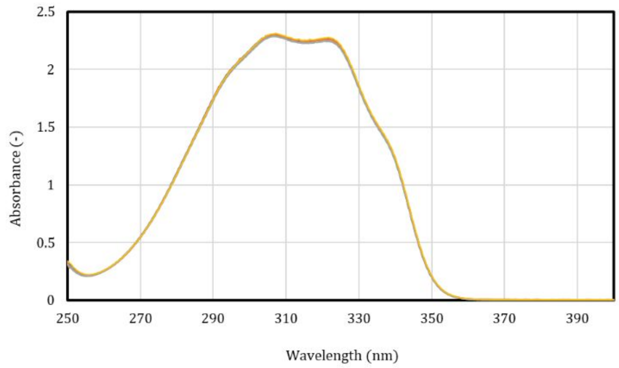

Absorbance (-)
Wavelength (nm)

b1)

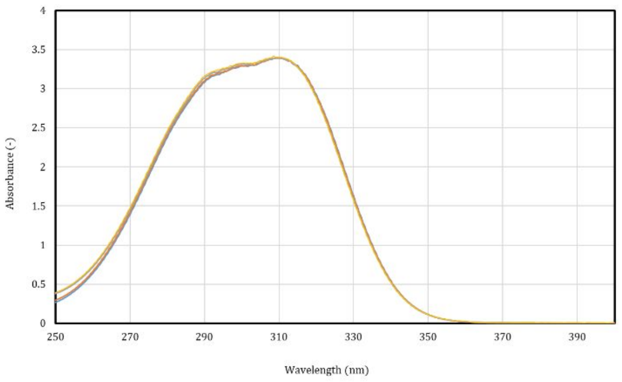

4
3.5
3
2.5
2
1.5
1
0.5
0
Absorbance (-)
250
270
290
310
330
350
370
390
Wavelength (nm)


b2)

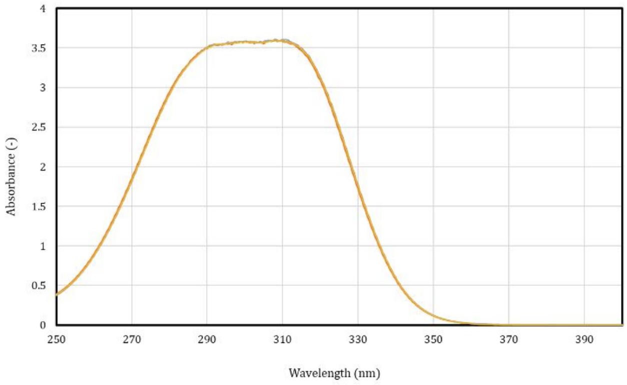

4
3.5
3
2.5
2
1.5
1
0.5
0
Absorbance (-)
250
270
290
310
330
350
370
390
Wavelength (nm)


b3)

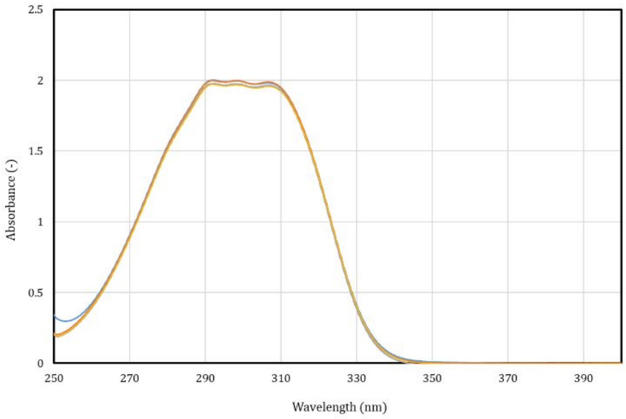

2.5
2
1.5
1
0.5
0
Absorbance (-)
250
270
290
310
330
350
370
390
Wavelength (nm)

c1)

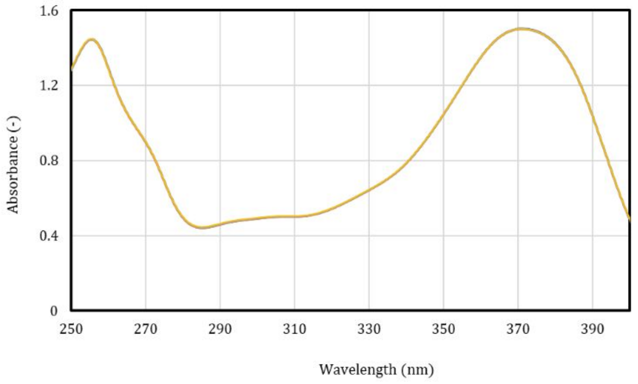

1.6
1.2
0.8
0.4
0
Absorbance (-)
250
270
290
310
330
350
370
390
Wavelength (nm)


c2)

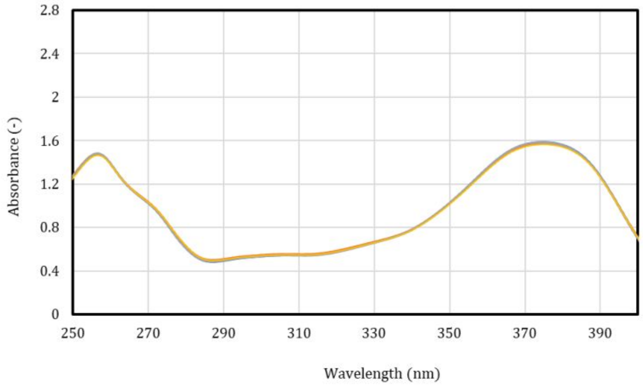

2.8
2.4
2
1.6
1.2
0.8
0.4
0
Absorbance (-)
250
270
290
310
330
350
370
390
Wavelength (nm)


c3)

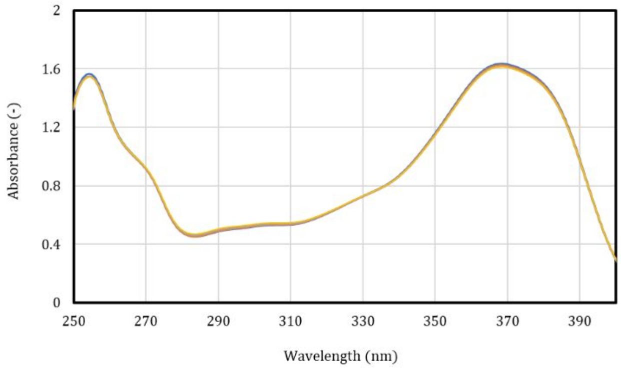

2
1.6
1.2
0.8
0.4
0
Absorbance (-)
250
270
290
310
330
350
370
390
Wavelength (nm)

d1)

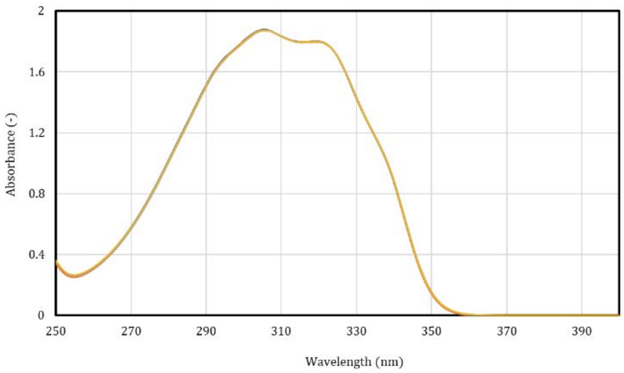


d2)

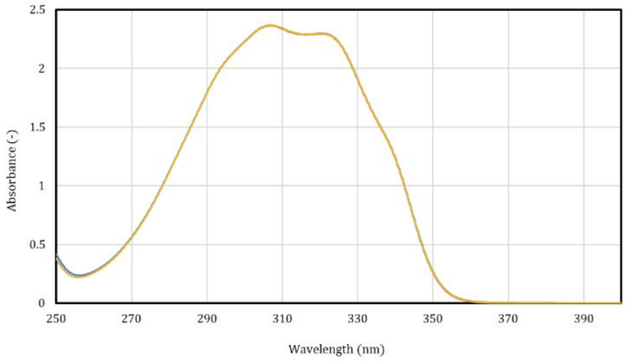


d3)

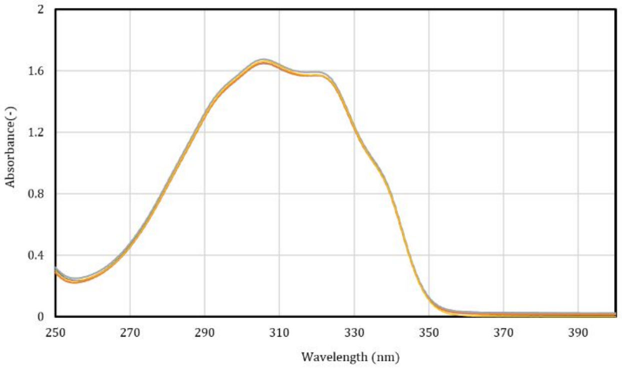


**Figure S3**. Ultraviolet spectra absorption graphs of (a) *trans*-polydatin, (b) *p*-coumaric acid, (c) quercetin, and (d) *trans*-resveratrol (1) methanol, (2) ethanol, and (3) ethyl acetate, measured (—) 0 h, (—) 24 h, (—) 48 h, and (—) 72 h after dissolution. Measurements were performed with 1 cm pathlength and blank subtraction.

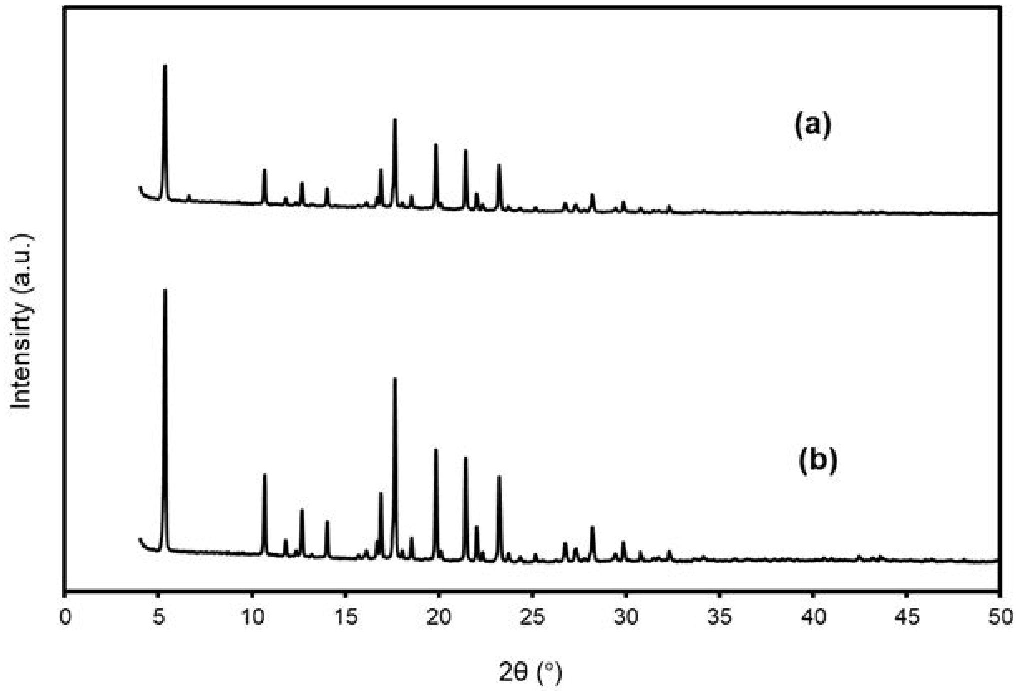


**Figure S4.** PXRD profiles of *trans*-polydatin: (a) raw material, and (b) recrystallized after solubilization in ethanol.

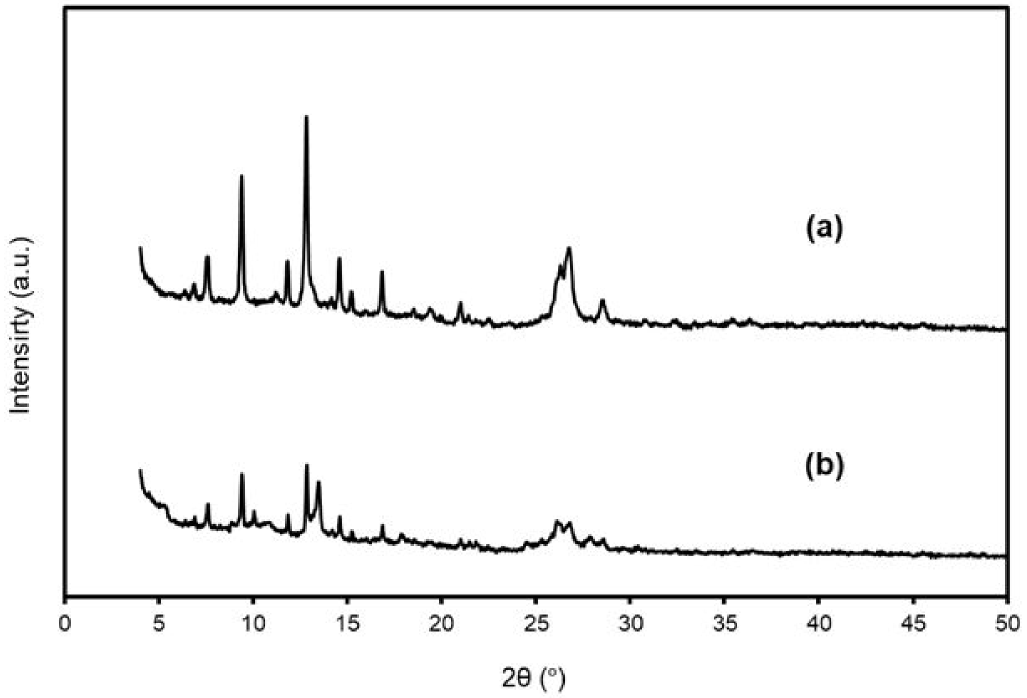


**Figure S5.** PXRD profiles of quercetin: (a) raw material, and (b) recrystallized after solubilization in ethanol.

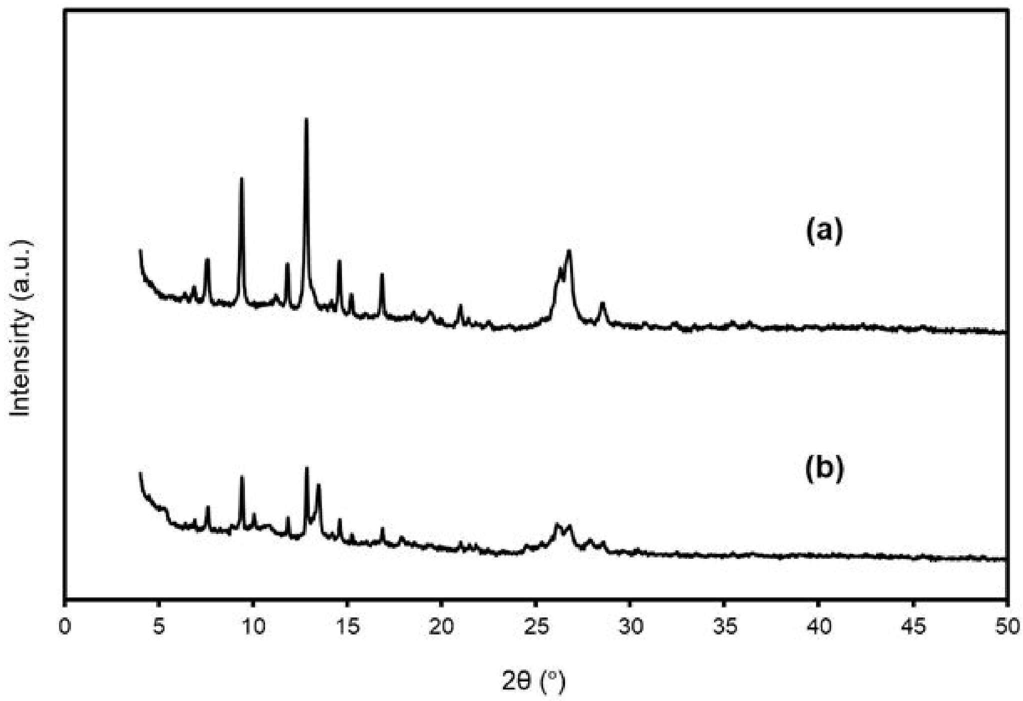


**Figure S6.** PXRD profiles of *trans*-resveratrol: (a) raw material, and (b) recrystallized after solubilization in ethanol.

a)

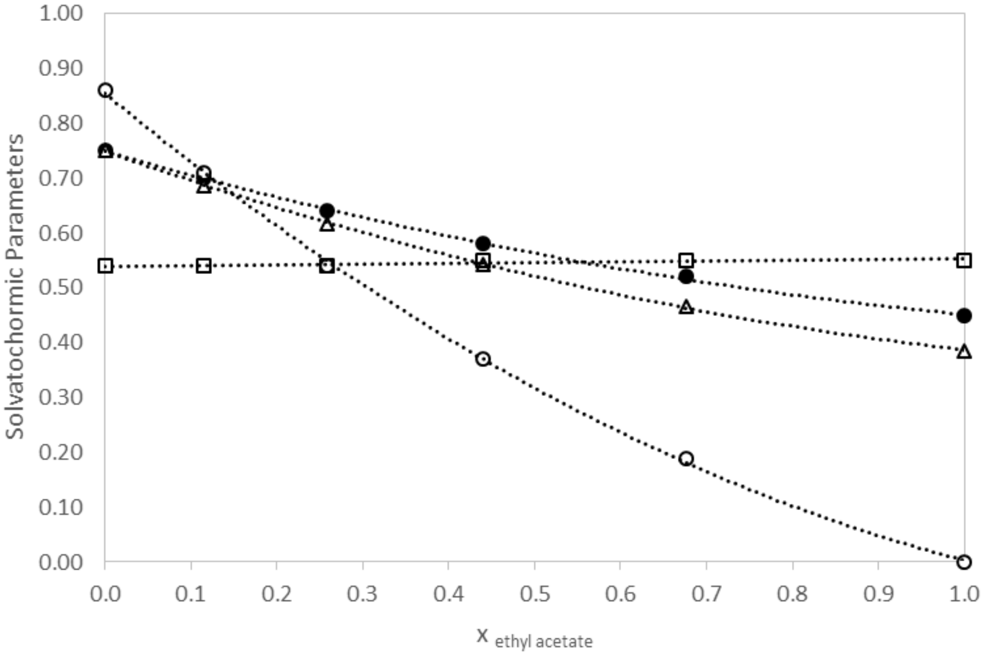


b)

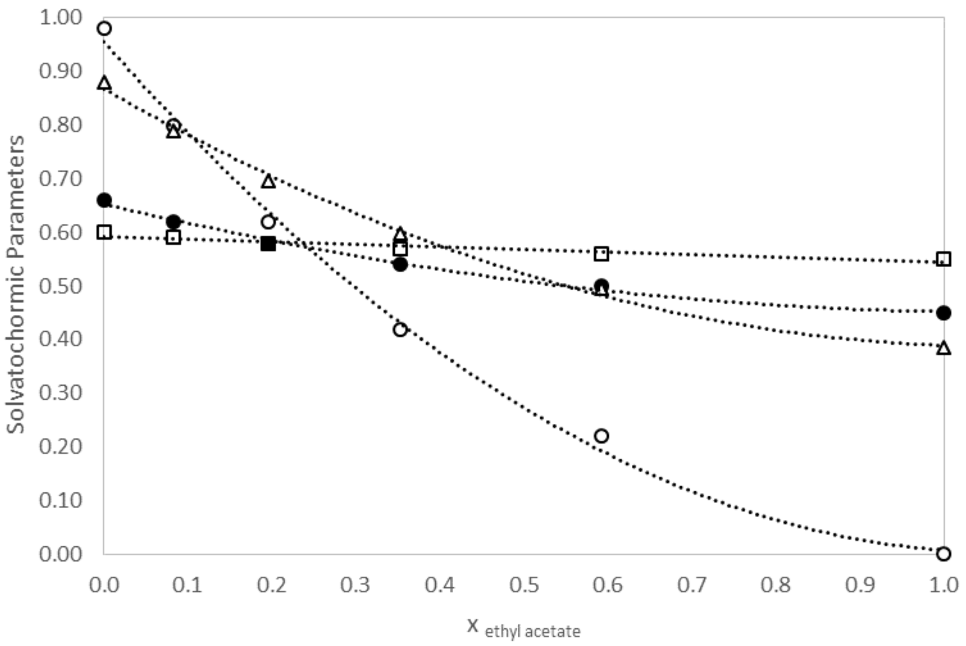


**Figure S7.** Solvatochromic parameters, (○) α, (●) β, (□) π*, and (△) Hildebrand solubility parameter, $\delta_H^2$, as a function of the mole fraction of the ethyl acetate ($x_{ethyl\ acetate}$) for the a) ethanol + ethyl acetate and b) methanol + ethyl acetate binary systems.

**Table S1** Abraham descriptors of studied polyphenols, obtained from the UFZ-LSER database [1] and Vilas-Boas *et al.* [2][a].

| Polyphenol | E | S | A | B | V |
|---|---|---|---|---|---|
| *p*-Coumaric acid | 1.472 | 1.138 | 0.29 | 0.877 | 1.429 |
| Quercetin | 2.68 | 2.2 | 1.92 | 1.4 | 1.9631 |
| *trans*-Resveratrol | 1.97 | 2.01 | 1.65 | 0.86 | 1.7391 |

[a] For *p*-coumaric acid

**Table S2** Abraham descriptors of tested solvents, obtained from the UFZ-LSER database [1]

| Polyphenol | E | S | A | B | V |
|---|---|---|---|---|---|
| Methanol | 0.28 | 0.44 | 0.43 | 0.47 | 0.3082 |
| Ethanol | 0.25 | 0.42 | 0.37 | 0.48 | 0.4491 |
| Ethyl acetate | 0.11 | 0.62 | 0 | 0.45 | 0.7466 |

**Table S3**. Mole fraction solubility of *trans*-polydatin ($x_p$), *p*-coumaric ($x_c$), quercetin ($x_q$), and *trans*-resveratrol ($x_r$) in ethanol + ethyl acetate, and mole fraction solubility of *trans*-resveratrol ($x_r$) and *p*-coumaric ($x_c$) in methanol + ethyl acetate at 298.2 *K* and 0.1 MPa, all expressed as a function of the mole fraction of ethyl acetate ($x_{ethyl\ acetate}$) in the binary solvent mixture[a].

| Ethanol + ethyl acetate | | | |
|---|---|---|---|
| $x_{ethyl\ acetate}$ | $x_p$ | $x_{ethyl\ acetate}$ | $x_c$ |
| 0.0000 | 0.0027 | 0.0000 | 0.0335 |
| 0.0983 | 0.0039 | 0.0983 | 0.0404 |
| 0.1974 | 0.0048 | 0.1974 | 0.0467 |
| 0.2981[1] | 0.0051 | 0.2981 | 0.0531 |
| 0.3968 | 0.0043 | 0.3968[1] | 0.0540 |
| 0.4913 | 0.0036 | 0.4913 | 0.0516 |
| 0.5960[2] | 0.0028 | 0.5960[2] | 0.0486 |
| 0.6967 | 0.0014 | 0.6967 | 0.0378 |
| 0.7976 | 0.0007 | 0.7976 | 0.0283 |
| 0.8974 | 0.0003 | 0.8974 | 0.0207 |
| 1.0000 | 0.0001 | 1.0000 | 0.0097 |
| $x_{ethyl\ acetate}$ | $x_q$ | $x_{ethyl\ acetate}$ | $x_r$ |
| 0.0000 | 0.0060 | 0.0000 | 0.0258 |
| 0.0983 | 0.0090 | 0.0983 | 0.0324 |
| 0.1974 | 0.0113 | 0.1974 | 0.0398 |
| 0.2981 | 0.0128 | 0.2981 | 0.0435 |
| 0.3968[1] | 0.0133 | 0.3968[1] | 0.0442 |
| 0.4913 | 0.0132 | 0.4913 | 0.0424 |
| 0.5960[2] | 0.0126 | 0.5960[2] | 0.0394 |
| 0.6967 | 0.0100 | 0.6967 | 0.0332 |
| 0.7976 | 0.0077 | 0.7976 | 0.0256 |
| 0.8974 | 0.0051 | 0.8974 | 0.0191 |
| 1.0000 | 0.0022 | 1.0000 | 0.0089 |
| Methanol + ethyl acetate | | | |

| $x_{ethyl\ acetate}$ | $x_r$ | $x_{ethyl\ acetate}$ | $x_c$ |
|---|---|---|---|
| 0.0000 | 0.0189 | 0.0000 | 0.0275 |
| 0.0966 | 0.0279 | 0.0972 | 0.0382 |
| 0.1992 | 0.0346 | 0.2009 | 0.0431 |
| 0.2881 | 0.0387 | 0.2957 | 0.0457 |
| 0.3965[1,2] | 0.0433 | 0.3967[1] | 0.0461 |
| 0.4979 | 0.0397 | 0.4918[2] | 0.0453 |
| 0.5960 | 0.0356 | 0.5911 | 0.0389 |
| 0.6956 | 0.0310 | 0.6906 | 0.0352 |
| 0.7966 | 0.0241 | 0.7965 | 0.0270 |
| 0.8978 | 0.0148 | 0.8990 | 0.0185 |
| 1.0000 | 0.0089 | 1.0000 | 0.0097 |

[a]The standard uncertainty of temperature is u(T) = 0.1 K, and that of pressure is u(P) = 1 kPa. Standard uncertainty for all mole fraction solubility values is $u(x_p\ x_c,\ x_q,\ x_r) = 0.0005$, and that for the mole fraction of acetate solvent is $u(x_{ethyl\ acetate})=0.0002$.
[1]Mole fraction of maximum solubility value.
[2]Mole fraction of máximum solubility excess.

**Table S4.** Solvatochromic parameters, α, β and π*and Hildebrand solubility parameter, $\delta_H^2$, as a function of the mole fraction of the ethyl acetate ($x_{ethyl\ acetate}$) for the ethanol + ethyl acetate and methanol + ethyl acetate binary systems.

| **ethanol + ethyl acetate** | | | | |
|---|---|---|---|---|
| $x_{ethylacetate}$ | π* | β | α | $\delta_H^2/1000$ |
| 0.0000 | 0.54 | 0.75 | 0.86 | 0.752 |
| 0.1156 | 0.54 | 0.70 | 0.71 | 0.686 |
| 0.2585 | 0.54 | 0.64 | 0.54 | 0.617 |
| 0.4396 | 0.55 | 0.58 | 0.37 | 0.544 |
| 0.6765 | 0.55 | 0.52 | 0.19 | 0.467 |
| 1.0000 | 0.55 | 0.45 | 0.00 | 0.386 |
| **methanol + ethyl acetate** | | | | |
| $x_{ethylacetate}$ | π* | β | α | $\delta_H^2/1000$ |
| 0.0000 | 0.60 | 0.66 | 0.98 | 0.880 |
| 0.0833 | 0.59 | 0.62 | 0.80 | 0.791 |
| 0.1951 | 0.58 | 0.58 | 0.62 | 0.697 |
| 0.3530 | 0.57 | 0.54 | 0.42 | 0.599 |
| 0.5926 | 0.56 | 0.50 | 0.22 | 0.495 |
| 1.0000 | 0.55 | 0.45 | 0.00 | 0.386 |

## References

[1] Helmholtz Centre for Environmental Research - UFZ, "UFZ-LSER database." Accessed: Oct. 08, 2025. [Online]. Available: https://web.app.ufz.de/compbc/lserd/public/start/.

[2] S. M. Vilas-Boas *et al.*, "Solid-liquid phase equilibrium of trans-cinnamic acid, p-coumaric acid and ferulic acid in water and organic solvents: Experimental and modelling studies," *Fluid Phase Equilib.*, vol. 521, 2020, doi: 10.1016/j.fluid.2020.112747.
